# Highly Active and Stable Large Catalase Isolated from a Hydrocarbon Degrading* Aspergillus terreus* MTCC 6324

**DOI:** 10.1155/2016/4379403

**Published:** 2016-01-19

**Authors:** Preety Vatsyayan, Pranab Goswami

**Affiliations:** ^1^Institute of Analytical Chemistry, Chemo- and Biosensors, University of Regensburg, 93053 Regensburg, Germany; ^2^Department of Biosciences and Bioengineering, Indian Institute of Technology Guwahati, Guwahati, Assam 781039, India

## Abstract

A hydrocarbon degrading* Aspergillus terreus* MTCC 6324 produces a high level of extremely active and stable cellular large catalase (CAT) during growth on* n*-hexadecane to combat the oxidative stress caused by the hydrocarbon degrading metabolic machinery inside the cell. A 160-fold purification with specific activity of around 66 × 10^5^ U mg^−1^ protein was achieved. The native protein molecular mass was 368 ± 5 kDa with subunit molecular mass of nearly 90 kDa, which indicates that the native CAT protein is a homotetramer. The isoelectric pH (*pI*) of the purified CAT was 4.2. BLAST aligned peptide mass fragments of CAT protein showed its highest similarity with the catalase B protein from other fungal sources. CAT was active in a broad range of pH 4 to 12 and temperature 25°C to 90°C. The catalytic efficiency (*K*_cat_/*K*_*m*_) of 4.7 × 10^8^ M^−1^ s^−1^ within the studied substrate range and alkaline pH stability (half-life, *t*_1/2_ at pH 12~15 months) of CAT are considerably higher than most of the extensively studied catalases from different sources. The storage stability (*t*_1/2_) of CAT at physiological pH 7.5 and 4°C was nearly 30 months. The haem was identified as haem b by electrospray ionization tandem mass spectroscopy (ESI-MS/MS).

## 1. Introduction

The importance of catalase for different industrial applications such as biosensors [1–5], therapeutics [6], and food and textile [7] is tremendously growing as revealed from the volume of publications on these areas since recent past. Catalase (EC 1.11.1.6) is a haem-containing antioxidant enzyme known for its ability to degrade hydrogen peroxide (H_2_O_2_) into water and oxygen [8]. The degradation occurs in two steps: the first step involves reduction of a H_2_O_2_ molecule into water with the concomitant oxidation of the catalase haem Fe^3+^ to oxyferryl species (Fe^4+^=O), while the second step involves oxidation of a second molecule of H_2_O_2_ into water and oxygen with the associated reduction of the oxyferryl species that regenerates the haem Fe^3+^.

Catalases are ubiquitous homotetrameric enzymes present in archaea, bacteria, fungi, plants, and animals. They are monophyletic in origin and are grouped into three clades: clade 1 in green algae and plants; clade 2 in archaea, bacteria, and fungi; and clade 3 in archaea, bacteria, fungi, and animals [9]. Clades 1 and 3 are composed of 55 to 69 kDa subunits, while clade 2 enzymes are formed by larger subunits of nearly 75 to 86 kDa [10]. In bacteria, the large catalases described are from* E. coli* (HPII or KatE),* Bacillus subtilis* (KatE),* Bacillus firmus* (KatE),* Mycobacterium avium* (KatE),* Pseudomonas putida* (CatC), and* Xanthomonas oryzae* (KatX) [11], whereas from fungi they are mostly reported from* Neurospora crassa* (Cat-1 and Cat-3) [12],* Penicillium vitale* [13],* Claviceps* (CPCAT1) [14], and* Aspergillus* species, namely,* A. nidulans* (CatA and CatB) [15–17],* A. fumigatus* (CatA and CatB) [18, 19], and* A. niger* (CatR) [20, 21]. The works on biochemical characterisation of large catalases from the* Aspergillus* species are mostly limited to* A. nidulans* [17],* A. fumigatus* [19], and* A. niger* [21].

**Scheme 1:**
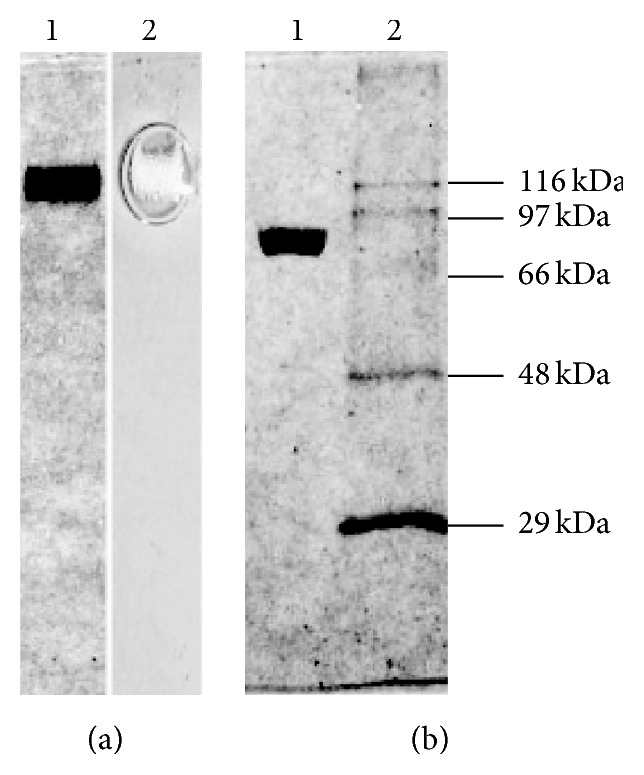
A schematic representation of metabolic machinery involved in hydrocarbon utilisation inside the cells of* A. terreus* that produces high level of H_2_O_2_ as byproduct which is then neutralised by CAT. CYP: cytochrome P450 monoxygenase and AOx: alcohol oxidase. (diagram not to scale).

Fungi,* A. terreus*, are widespread and abundant in terrestrial ecosystem due to their capability to utilise a wide variety of carbon substrates for growth and survival [22–24]. This makes them a potential target for search of novel biocatalysts (enzymes) involved in metabolic machinery of these diverse susbstrates. We already made detailed investigation on enzymes involved in metabolic machinery involved in the hydrocarbon utilisation in* A. terreus* MTCC 6324 isolated from oil fields of Assam (India) [23, 25–27]. The isolated enzymes cytochrome P450 monoxygenase (CYP) and alcohol oxidase (AOx) were used succesfully for their biosensor and industrial applications [28, 29]. However, as shown in Scheme 1, the metabolic utilisation of hydrocarbon produces a huge amount of H_2_O_2_ as biproduct inside the cells which may be of damage to cell. We expected the presence of some highly efficient H_2_O_2_ metabolising enzyme inside the cells to combat this oxidative stress for cell survival. This led us to identify, purifiy, and characterise a large catalse (CAT) from this fungal strain which showed excellent catalytic efficiency and stability. The work on utilisation of this CAT protein for fabrication of a highly stable and sensitive biosensor has been published [30]. In this work, we focus on the isolation, purification, characterisation, and establishment of the potentially interesting properties of this novel CAT from* A. terreus* which has lot of untapped potential for future applications.

## 2. Materials and Methods

### 2.1. Organism and Culture Conditions

The fungal strain* A. terreus* used in this investigation is a stock culture of Microbial Type Culture Collection (MTCC), Chandigarh, with accession number 6324. The culture conditions and maintenance of* A. terreus* MTCC 6324 used in this study were described elsewhere [25]. Briefly, the organism was cultivated in 500 mL Erlenmeyer flasks containing 2%* n*-hexadecane/glucose in 50 mL of basal medium [31]. The pH of the medium was adjusted to 5.8 and the flasks were incubated at 28°C under static condition. The organism was maintained on fungal agar slants with periodic transfer to a new slant following each 15 days.

### 2.2. Purification of CAT

The fungal mycelia were harvested from the early stationary phase of growth (72 hours) and were disrupted at 30 kpsi using mechanical cell disruptor (Constant Systems, UK) by following the procedure described by Kumar and Goswami [25]. The disrupted cell homogenate was centrifuged successively at 10,000 ×g for 10 min and 20,000 ×g for 20 min to pellet undisrupted cell mass and light mitochondrial fractions. The supernatant thus obtained was initially adjusted to 50% (NH_4_)_2_SO_4_ saturation to precipitate the contaminating proteins. After removing the precipitated protein by centrifugation at 10,000 ×g for 30 min, the supernatant was finally adjusted to 80% (NH_4_)_2_SO_4_ saturation to precipitate the CAT protein and centrifuged at 10,000 ×g for 30 min. The pellet was resuspended in 50 mM sodium phosphate buffer (SPB) containing 1 M (NH_4_)_2_SO_4_ and loaded on a Phenyl Sepharose 6 Fast Flow hydrophobic interaction chromatography (HIC) column (1.5 × 25 cm) connected to fast pressure liquid chromatography (FPLC) (AKTA prime plus, GE), preequilibrated with 50 mM SPB (pH 7.5) containing 1 M (NH_4_)_2_SO_4_. The fractions were eluted from the column using 50 mM SPB (pH 7.5) containing a decreasing step gradient of (NH_4_)_2_SO_4_ (0.75 M, 0.5 M, and 0.25 M). The final fraction was eluted with 50 mM SPB (pH 7.5). Fractions containing CAT activity were pooled, dialysed, concentrated, and then loaded on HiPrep Sephacryl S-300 HR (1.6 × 60 cm, 50 *μ*m) size exclusion chromatography (SEC) column preequilibrated with 50 mM SPB (pH 7.5) to be eluted with the same buffer and flow rate. CAT-containing fractions were pooled and concentrated. The lyophilised purified CAT was stored at −20°C and was used for further characterisation after resuspension in 50 mM SPB (pH 7.5).

### 2.3. Molecular Weight Determination and Electrophoresis

Molecular weight of purified native CAT was determined by SEC (HiPrep Sephacryl S-300 HR, 1.6 × 60 cm, 50 *μ*m) using the FPLC (AKTA prime plus, GE). The gel filtration molecular weight standards used were thyroglobulin (669 kDa), apoferritin (443 kDa), *β*-amylase (200 kDa), alcohol dehydrogenase (150 kDa), and carbonic anhydrase (29 kDa) (Sigma).

Native and SDS-PAGE analyses of the purified CAT protein were done following the method of Laemmli [32] using 7% and 10% separating gels, respectively, with 5% stacking gel and thickness of 0.75 mm. The gels were stained with Coomassie brilliant blue (CBB) R 250 (Merck, India). Zymogram analysis of the native PAGE was done by incubating the gel in 10 mM H_2_O_2_ for 10 min. The SDS-PAGE protein markers used were carbonic anhydrase (bovine erythrocyte, 29 kDa), fumarase (porcine heart, 48.5 kDa), serum albumin (bovine, 66 kDa), phosphorylase b (rabbit muscle, 97.4 kDa), and *β*-galactosidase (*E. coli*, 116 kDa) (Sigma). Glycostaining of the protein band in SDS-PAGE was done following the method of Segrest and Jackson [33] using periodic acid Schiff (PAS) reagent.

The* pI* of CAT was determined by isoelectric focusing following the method described elsewhere [26].

The haem from the purified CAT isolated by 2-butanone/HCl method [34] was characterised by electrospray ionization tandem mass spectroscopy (ESI-MS/MS) (Q-TOF Premier, Waters) following the method of Sana et al. [35] and Fourier transform infrared spectroscopy (FTIR) (Perkin-Elmer).

### 2.4. Peptide Mass Fingerprinting

The protein identification work was carried out at ProtTech, Inc. (USA) by using the nanoliquid chromatography-tandem mass spectroscopy (LC-MS/MS) peptide sequencing technology following the method described elsewhere [26].

### 2.5. Enzyme Assay and Kinetics

The CAT activity was assayed by the method of Beers and Sizer [36] with a partial modification. The standard reaction mixture for the assay contained 50 mM SPB (pH 7.5) and 4.9 mM H_2_O_2_ (*ε*_240_ = 43.6 M^−1 ^cm^−1^). Initial reaction rate was determined spectrophotometrically (Cary 100Bio, Varian) by measuring the decrease of absorbance at 240 nm. One unit (U) of enzyme activity is the amount of enzyme that consumes 2 *μ*mol H_2_O_2_ in one minute at room temperature. The substrate saturation kinetics was measured with H_2_O_2_ concentrations in a range of 0.05 to 75 mM and enzyme preparation bearing specific catalase activity 30.74 × 10^5^ U mg^−1^ protein. The apparent Michaelis-Menten constant (*K*_*m*_) for H_2_O_2_ was calculated from the Lineweaver-Burk plot.

### 2.6. pH and Temperature Optima and Stability of CAT

To measure pH optima, the enzyme reactions were carried out in different pH buffers, namely, trisodium citrate (pH 2.5 to 4), sodium acetate (pH 5), sodium phosphate (pH 6 to 7.5), tris (pH 8), ethanolamine (pH 9), and sodium phosphate (pH 10 to 12.5), each at a concentration of 50 mM. For pH stability measurement the enzyme samples were incubated for different time periods in the aforementioned pH buffers at 4°C and the residual CAT activity was determined at room temperature in SPB (50 mM, pH 7.5). The temperature optima were determined by carrying out the enzyme reactions at different temperatures and the CAT activity was measured spectrophotometrically. For determination of temperature stability, enzyme samples were incubated at different temperatures (4°C to 80°C) in circulating water bath (Julabo SW22, Germany) for different time periods followed by measurement of residual activity of CAT at room temperature in SPB (50 mM, pH 7.5).

The half-life (*t*_1/2_) was calculated from the value of 0.693/*k*, where “*k*” is the first-order rate constant. The value of “*k*” was calculated from the slope of a curve obtained by plotting ln⁡*a*_0_/*a*_*t*_ versus time (*t*), where *a*_0_ is the initial CAT activity and *a*_*t*_ is the residual CAT activity obtained by incubating the CAT for different time periods at said pH or temperature.

Protein was estimated by Bradford's method using bovine serum albumin as standard [37] and carbohydrate was estimated by anthrone method using glucose as standard [38].

## 3. Results and Discussion

A very high level of catalase activity was detected in the vegetative cells of* A. terreus* during its growth on* n*-hexadecane (2% v/v) as the sole source of carbon for growth. A total activity of 17 × 10^6^ U gm^−1^ dry cell mass was obtained in the cells harvested from the late exponential phase (90 h) of growth in* n*-hexadecane substrate. This activity in the corresponding glucose grown cells was 4.25 × 10^6^ U gm^−1^ dry cell mass. The average catalase activity from late log phase to late exponential phase was 9.5 × 10^6^ U gm^−1^ dry cell mass in* n*-hexadecane grown cells. The overall activity decreased in the stationary phase of the growth of fungus. Thus, around 4-fold increased catalase activity was recorded in the late exponential phase of hydrocarbon grown cells of* A. terreus* when compared to glucose grown cells of the same strain. Such increased level of catalase activity in the* n*-hexadecane grown cells can be linked to the metabolism of this hydrocarbon substrate in the cells of fungal mycelia (Scheme 1). The initial oxidation of* n*-hexadecane by* A. terreus* produces hexadecanol [23], which is further oxidised by a long-chain alcohol oxidase with concomitant generation of H_2_O_2_ as byproduct [25, 26]. It is expected that the high level of catalase produced in the cells of* A. terreus* rapidly neutralises the harmful H_2_O_2_ continuously generated as byproduct in the cells during metabolism of the hydrocarbon substrate. Thus, the induction of such high level of cellular large catalase (as identified by biochemical studies discussed later in this section, abbreviated as CAT) can be credited as the cellular defence mechanism against the oxidative stress of H_2_O_2_ generated during metabolism of* n*-hexadecane. The fact that large catalases have been reported to be less sensitive to oxidative damage by H_2_O_2_ also supports the given assumption [12, 21]. Although the induction of peroxide degrading enzyme genes (katG encoding catalase-peroxidase) has been reported from polycyclic aromatic hydrocarbon degrading* Mycobacterium* sp. [39], any such induction of cellular large catalase from the hydrocarbon degrading cells of fungus is not reported before. Notably, except the level of induction, other properties of the CAT from* n*-hexadecane grown cells (as described later in this section) were alike to the one produced in the glucose grown cells (results not separately shown).

**Figure 1:**
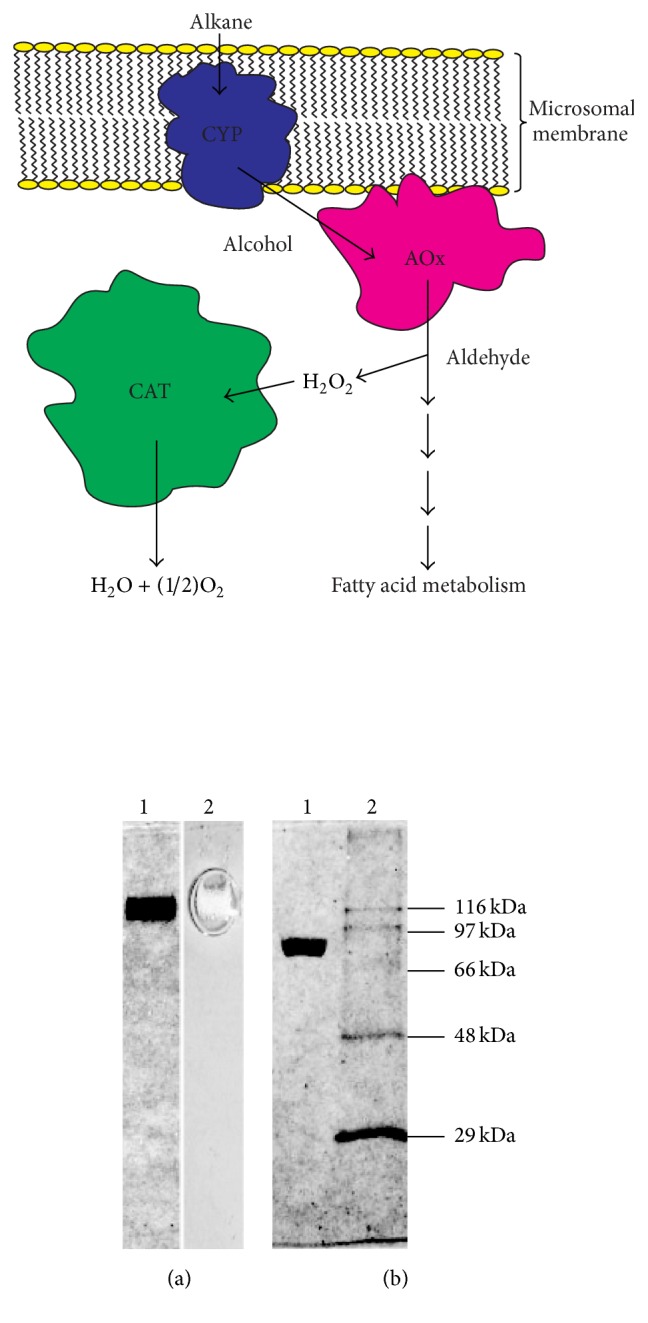
PAGE analysis of purified CAT. (a) Native PAGE: lane 1, CBB staining of CAT and lane 2, activity staining of CAT (loaded 10 *μ*g purified protein). (b) SDS-PAGE: lane 1, CBB staining of CAT protein and lane 2, CBB staining of standard molecular markers.

**Figure 2:**
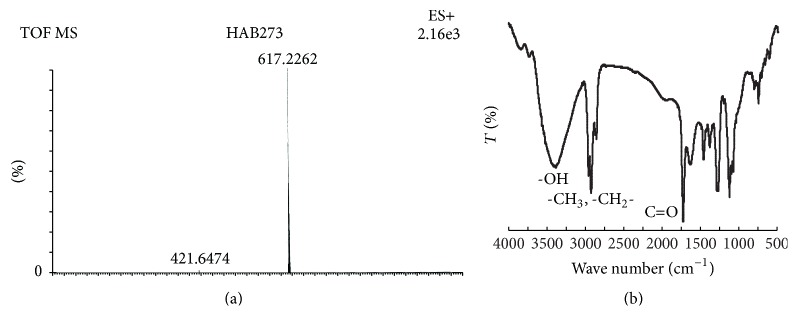
(a) MS spectrum of the extracted haem from the native purified CAT. The sample was run in the ESI positive mode. Details of the experiment and results are discussed in the paper. (b) FTIR spectra of the isolated haem. Peaks corresponding to the representative functional groups of haem are shown in the figure.

CAT was isolated and purified successively by differential centrifugation, (NH_4_)_2_SO_4_ precipitation, HIC, and SEC. A 160-fold purification with specific activity of 66 × 10^5^ U mg^−1^ protein with an overall yield of 47% in comparison to the original crude extract was achieved (Table 1). The homogeneity of the purified protein was demonstrated by single protein band in native PAGE (Figure 1(a), lanes 1 and 2). The molecular weight of the CAT determined by SDS-PAGE of the purified protein sample (Figure 1(b), lanes 1 and 2) was 90 ± 2 kDa. The native protein molecular mass determined by SEC was 368 ± 5 kDa (see Figure S1 of the Supplementary Material available online at http://dx.doi.org/10.1155/2016/4379403), which indicates that the native CAT protein is a homotetramer with subunit molecular mass ~90 kDa and thus belongs to the clade 2 of catalases constituting larger subunits (>80 kDa). The purity of the CAT was further demonstrated by the high optical purity ratio (Reinheitszahl or* Rz*, *A*_405_/*A*_280_) of 0.8 ± 0.05 (*A*_405_ is Soret band typical for catalases [40]) (Figure S2). A distinct but low intensity peak at around 570 nm in the same spectrum is attributed to the presence of a low concentration of inactive form of CAT [41]. The inactivation of CAT may have resulted from the addition of dithiothreitol in disrupted cell homogenate following the procedure described by Kumar and Goswami [25]. The characteristic charge-transfer band (reflecting the heme Fe^(III)^ to Tyr axial ligand electronic structure in catalase) at ca. 630 nm also appears to be masked by this peak. The presence of this peak also provides the reason for* Rz* (*A*_405_/*A*_280_) of 0.8 ± 0.05 (less than 1), despite the fact that the protein was purified to homogeneity as depicted from the fold purification (Table 1) and absence of any other contaminating band in SDS- and native PAGE. The isoelectric pH (*pI*) determined by isoelectric focusing of the purified CAT was found to be 4.2 ± 0.1. The CAT protein was identified as glycoprotein based on the PAS staining of the protein band in SDS-PAGE. The protein to carbohydrate ratio of the purified catalase was 3.33. The haem was isolated from the purified CAT and the molecular mass of 616 of the isolated haem was determined by ESI-MS/MS (Figure 2(a)), which is similar to the reported molecular mass of haem b [35]. The isolated haem was further characterised for its functional groups -OH, -CH_3_, -CH_2_-, and -C=O by FTIR spectra (Figure 2(b)). Many variants of haem, namely, haem-b, -d, and derivatives of haem have been reported in catalases. Notably, the presence of only haem d in large catalases from* Aspergillus* has been described so far [21]. Thus, this finding on the presence of haem b in the large catalase from* A. terreus* is interesting and warrants further investigation to understand the exact role of these two types of haem (b and d) in the large catalases, though haem in general is known as the catalytic centre of these redox enzymes.

BLAST alignment of peptide mass fragments from the SDS-PAGE separated protein band of the purified CAT showed its highest similarity with the catalase B precursor protein from* A. terreus* NIH 2624 (Accession code: XP_001216098, http://www.ncbi.nlm.nih.gov/). Figures S3 and S4 show the LC-MS/MS tandem mass spectra of proximal haem binding and tetramer interface domains of the CAT protein, respectively. The presence of fully conserved positions for tyrosine (Y) residue in the proximal haem binding domain is evident from the multiple sequence alignment of proximal haem binding residues of catalases from different source organisms (Table 2). It is known that phenolic side chain of the conserved tyrosine acts as the fifth haem iron ligand, the other four being the nitrogens of the porphyrin ring. The overall sequence of CAT was found to be conserved among the large catalases from bacteria and fungi while being distinct from smaller catalases from bacteria and higher organisms.

Catalases in general are not known to exhibit Michaelis-Menten kinetics over the entire substrate range (from mM to molar concentrations of H_2_O_2_) and they usually have the two-step nature of the catalytic reaction [21]. The large catalase from* A. terreus* showed increased catalytic activity with increasing H_2_O_2_ concentrations (within 0.05 to 75 mM H_2_O_2_). The velocity of enzymatic reaction showed a Michaelis-Menten-type dependence on substrate concentration with a saturation of catalytic activity at around 75 mM. The apparent *K*_*m*_ of 14.15 mM was calculated from the Lineweaver-Burk plot with a linear equation, *y* = 4.265*x* + 0.301, and correlation coefficient (*R* ^2^) of 0.9949. The apparent *K*_*m*_ of CAT from* A. terreus* correlates well with the apparent *K*_*m*_ (21.7 mM) of large catalase (Cat-1) of* N. crassa* at H_2_O_2_ concentrations below 100 mM [12]. However, at molar concentrations of H_2_O_2_, a 10 times higher apparent *K*_*m*_ was reported for* N. crassa* Cat-1. The living organisms in nature are seldom exposed to molar concentrations of H_2_O_2_; thus the studies with such high concentrations of H_2_O_2_ were consciously avoided in this work. However, the inhibition studies of CAT and its kinetics at molar concentration of H_2_O_2_ are subject of future investigations. A high catalytic efficiency (*K*_cat_/*K*_*m*_) of 4.7 × 10^8^ M^−1^ s^−1^ was deduced from the catalytic turnover number (*K*_cat_) of 6.65 × 10^6^ s^−1^ and apparent *K*_*m*_ of the CAT (for the above-mentioned range of H_2_O_2_ concentrations). The high catalytic efficiency reported by us is only comparable to the durable Cat-1 of* N. crassa* (4.11 × 10^8^ M^−1^ s^−1^, within 100 mM H_2_O_2_) [12], which is stated to be produced to combat the oxidative stress developed during the conidia formation. This correlates well with our previous assumption for production of high level of extremely efficient CAT in* A. terreus* to combat the oxidative stress during assimilation of long-chain hydrocarbons in the cell.

**Figure 3:**
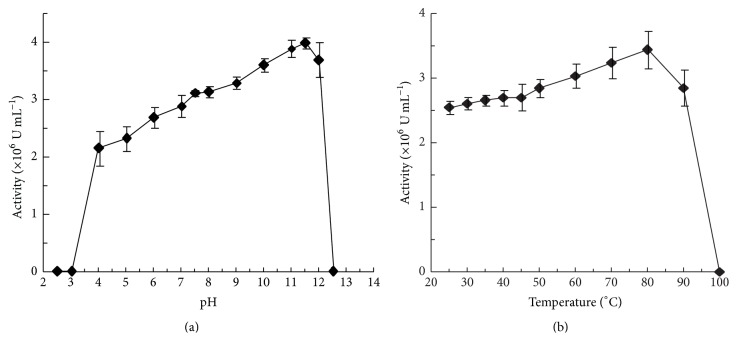
CAT activity as a function of pH (a) and temperature (b). The different pH buffers (each at a concentration of 50 mM) were trisodium citrate (pH 2.5 to 4), sodium acetate (pH 5), sodium phosphate (pH 6 to 7.5), tris (pH 8), ethanolamine (pH 9), and sodium phosphate (pH 10 to 12.5). Each datum point represents the average of the analysis of triplicate values.

The large catalase from* A. terreus* did not show any specific pH or temperature optimum for maximum activity but was active throughout the broad range of pH 4 to 12 and temperatures 25°C to 90°C (Figures 3(a) and 3(b)). However, a variation in CAT activity was observed within this range, where the activity increased with the increasing pH and temperature. The CAT activity was drastically lost outside this pH range (pH 4 to 12) and when boiled. Although CAT was active throughout the mentioned pH range, the maximum stability of CAT (measured as retention of its catalytic activity) was recorded at around physiological pH (pH 7.5) with the *t*_1/2_ of ~30 months. CAT was also found to be exceptionally stable at alkaline pH. The *t*_1/2_ of the CAT at pH 12 was ~15 months. The stability of CAT diminished steadily with the increasing temperature from 25°C to 80°C with *t*_1/2_ of 54 days and 42 min at 25°C and 80°C, respectively. The maximum stability of CAT was recorded when stored at 4°C and pH 7.5, as more than 98% of initial catalase activity was retained even after one month and a *t*_1/2_ of nearly 30 months was calculated. Although the broad pH and temperature optima and many other biochemical properties discussed before in this section such as acidic* pI*, glycoprotein nature, and binding to hydrophobic column of the isolated CAT from* A. terreus* are similar to the widely studied large catalases reported from other* Aspergillus* strains [17, 19, 21], certain properties of CAT were found to be unique and not reported to date from these sources. The catalytic efficiency and stability near extreme alkaline pH of CAT are considerably higher than most of the extensively studied catalases from different sources [17, 21, 42, 43]. The thermal stability of CAT at 80°C is considerably high [21] and is only comparable with the catalase from thermophilic origin [44], Cat-1 from* N. crassa* [12], and HPII of* E. coli* [45]. High storage stability at 4°C and 25°C (room temperature) is also interesting feature of the isolated CAT. The stability of CAT at extreme alkaline pH (pH 12) was intriguing and drove us to investigate the reasons behind such behaviour (not part of this report). The early spectroscopic and electrochemical studies of CAT at different pH conditions (from pH 3 to 12) showed that acidic conditions induced the dissociation of haem from CAT whereas the association of haem with protein matrix was quite stable at basic pH values [46]. These findings provided some of the reasons for the higher activity and stability of CAT at alkaline pH in this study and sudden loss of activity at pH 3. Thus, besides the sequence conservation and similarities in various biochemical properties of CAT from* A. terreus* with other large catalases from bacterial and fungal sources, understanding the differences in some of the specific behaviours on the basis of structure-function relationship studies is always a possibility. Such variations in protein chemical properties such as specific activities, reaction velocities, and stability among a large number of catalases bearing extensive sequence similarities have been reported earlier also by other research groups [21], further supporting our claim for the requirement of separate studies for different catalases from diverse sources. The remarkable activity and stability of CAT under broad thermal and pH conditions are construed as attractive properties for potential applications in areas like biosensors as well as therapeutics, textile, and food industries where the enzyme is usually required to work in diverse pH and temperature conditions for long periods. The biosensing application of CAT with a considerably high sensitivity and stability is already established in one of our investigations [30], fortifying the potential of this large catalase from* A. terreus* in areas where catalytic efficiency and stability of protein are major concerns.

## 4. Conclusions

A considerably high level of catalase activity is reported inside the cells of* A. terreus* (originally isolated from the oil fields of Assam, India) when grown on hydrocarbon substrate. The reason for this high level of catalase activity is attributed to the necessity to neutralise the high level of H_2_O_2_ produced as a byproduct of the metabolic machinery for hydrocarbon assimilation inside the cells. Although the isolated large catalase from* A. terreus* shares many functional similarities with other large catalases reported from different microbial sources, its remarkable stability at alkaline pH and catalytic efficiency are intriguing. These properties establish the potential of this large catalase for biosensing and other industrial applications and also require further investigations to understand the structure-function relationship behind such specificities.

## Supplementary Material

The following supplementary material provides the detailed and supporting data for the findings discussed in the paper.

## Figures and Tables

**Table 1:** Purification table for CAT.

Fractions	Activity (×10^5^ U mL^−1^)	Protein (mg mL^−1^)	Specific activity (×10^5^ U mg^−1^)	Fold purification	Yield %
crude extract	1.2 ± 0.5	2.9 ± 0.8	0.41 ± 0.10	1.0	100
sup-1^a^	1.7 ± 0.3	1.8 ± 0.5	0.94 ± 0.20	2.3	88.6
pellet-1^a^	2.7 ± 0.8	5.4 ± 1.0	0.50 ± 0.09	1.2	10.8
sup-2^b^	2.3 ± 0.4	1.4 ± 0.3	1.64 ± 0.60	4.0	75.0
pellet-2^b^	0.3 ± 0.1	7.2 ± 1.2	0.04 ± 0.01	0.1	12.4
50% pellet^c^	2.4 ± 0.7	7.2 ± 1.9	0.33 ± 0.10	0.8	10.1
80% pellet^c^	3.3 ± 1.2	1.4 ± 0.5	2.36 ± 0.50	5.8	62.5
HIC purified^d^	14.7 ± 1.4	0.5 ± 0.1	29.40 ± 1.42	71.7	54.4
SEC purified	39.6 ± 2.0	0.6 ± 0.1	66.00 ± 3.97	160.9	47.0

^a^sup-1 and pellet-1 are supernatant and pellet of 10,000 ×g centrifugation.

^b^sup-2 and pellet-2 are supernatant and pellet of 20,000  ×g centrifugation.

^c^50% and 80% pellet are (NH_4_)_2_SO_4_ precipitated fractions.

^d^HIC purified fraction was eluted at 250 mM (NH_4_)_2_SO_4_ concentration.

Each value represents the mean ± standard error at *p* < 0.05.

**Table 2:** Multiple sequence alignment of amino acid residues of proximal haem binding domain of CAT with other known catalases.

Catalase source organism	Amino acid sequence	Accession code
*Aspergillus terreus* MTCC 6324	R L F S **Y** L D T Q	This work
*Aspergillus terreus* NIH 2624	R L F S **Y** L D T Q	XP_001216098.1
*Aspergillus niger *	R L F S **Y** L D T Q	XP_001388621.1
*Aspergillus nidulans* FGSC A4	R L F S **Y** L D T Q	XP_682608.1
*Aspergillus fumigatus* Af293	R L F S **Y** L D T Q	XP_748550.1
*Penicillium marneffei* ATCC 18224	R L F S **Y** L D T Q	XP_002153601.1
*Neurospora crassa* OR74A	R L F S **Y** L D T Q	XP_957826.1
*Pseudomonas stutzeri* A1501	R L F S **Y** A D T Q	YP_001174038.1
*Escherichia coli* SE11	R L F S **Y** T D T Q	YP_002293177.1
*Mycobacterium vanbaalenii* PYR-1	R L F S **Y** L D T Q	YP_954009.1
*Candida dubliniensis* CD36	R L F S **Y** A D T H	dbj|BAD77826.1
*Arabidopsis thaliana *	R V F S **Y** A D T Q	emb|CAA64220.1
*Rattus norvegicus *	R L F A **Y** P D T H	NP_036652.1
*Bos taurus *	R L F A **Y** P D T H	NP_001030463.1
*Homo sapiens *	R L F A **Y** P D T H	NP_001743.1
