# Supplementary material for: Highly Active and Stable Large Catalase Isolated from a Hydrocarbon Degrading Aspergillus terreus MTCC 6324
Source: Enzyme Res. 2016 Jan 19;2016:4379403. doi: 10.1155/2016/4379403 (PMC4807065; doi:10.1155/2016/4379403)
Supplement: Supplementary file 1 — The following supplementary material provides the detailed and supporting data for the findings discussed in the paper. [file 4379403.f1.pdf]

Supplementary material

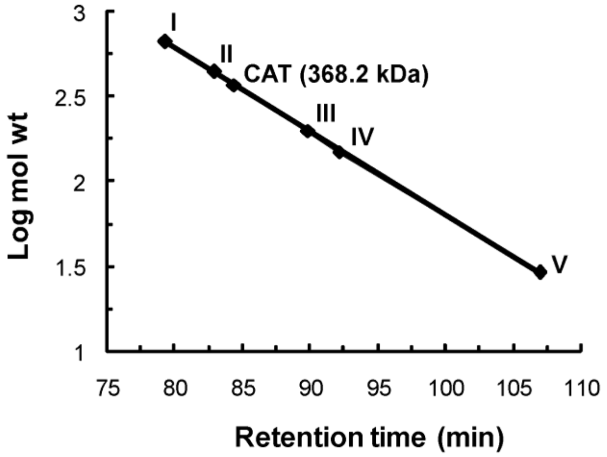

Fig. S1 Determination of molecular weight of native CAT isolated from *A. terreus* by SEC column (Hiprep Sephacryl S-300 HR, 1.6×60 cm, 50 µm, GE). Molecular weight standards were: I. thyroglobulin (669 kDa), II. apoferritin (443 kDa), III. β-amylase (200 kDa), lV. alcohol dehydrogenase (150 kDa) and V. carbonic anhydrase (29 kDa) (Sigma)

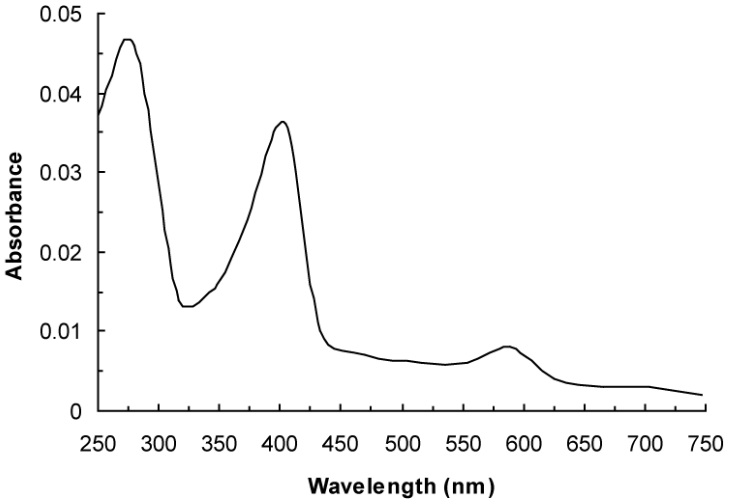

Fig. S2 UV-Vis spectra of purified CAT. The protein concentration was 15 µg ml^{-1} in 50 mM SPB (pH 7.5). The data was recorded at a scan rate of 100 nm min^{-1}

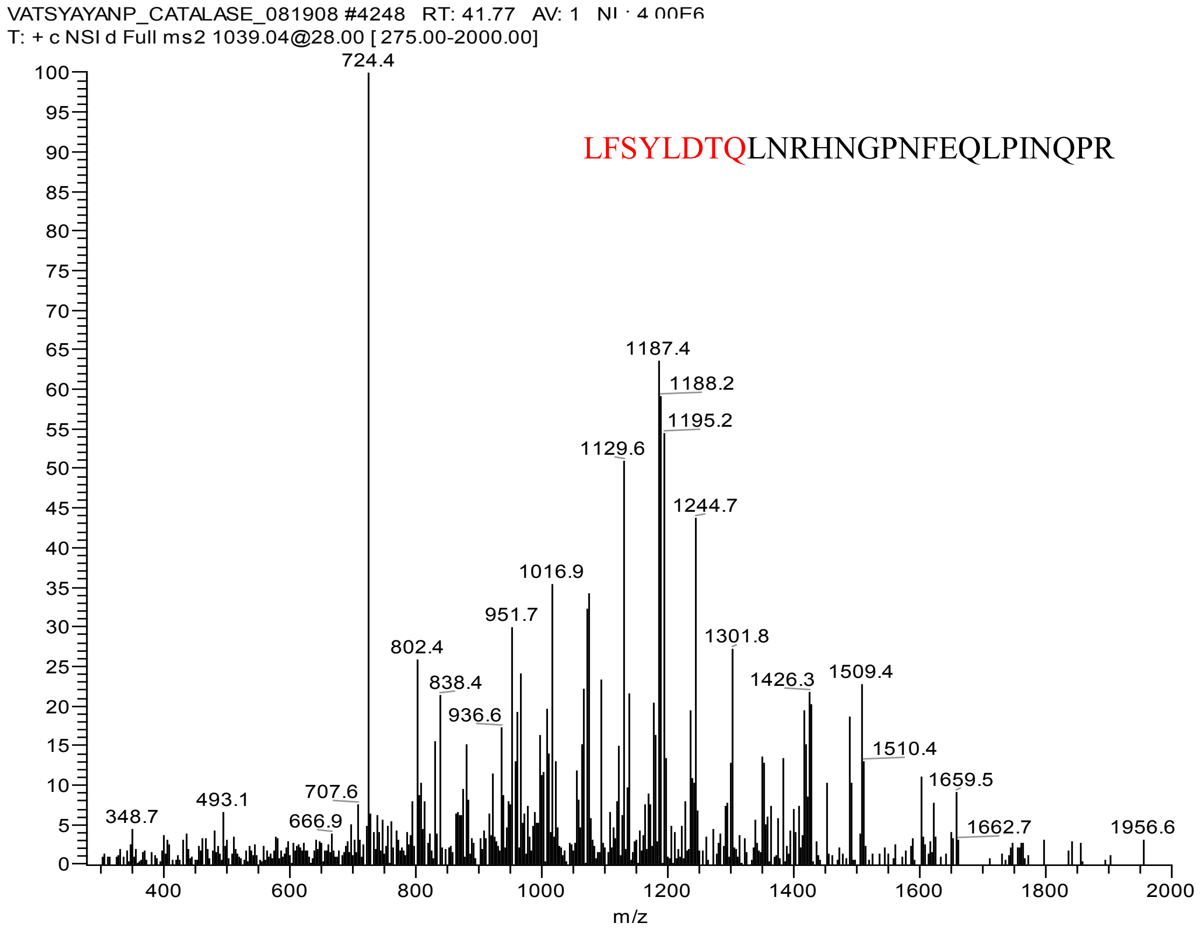

Fig. S3 LC-MS/MS tandem mass spectra of "LFSYLDTQLNRHNGPNFEQLPINQPR" peptide. The eight amino acid sequences from left belong to the proximal heme binding domain of the CAT. The first residue "R" in the left is missed here due to tryptic digestion, which is however, identified by the overlapping of spectra. The detailed description of proximal heme binding domain is given in the manuscript

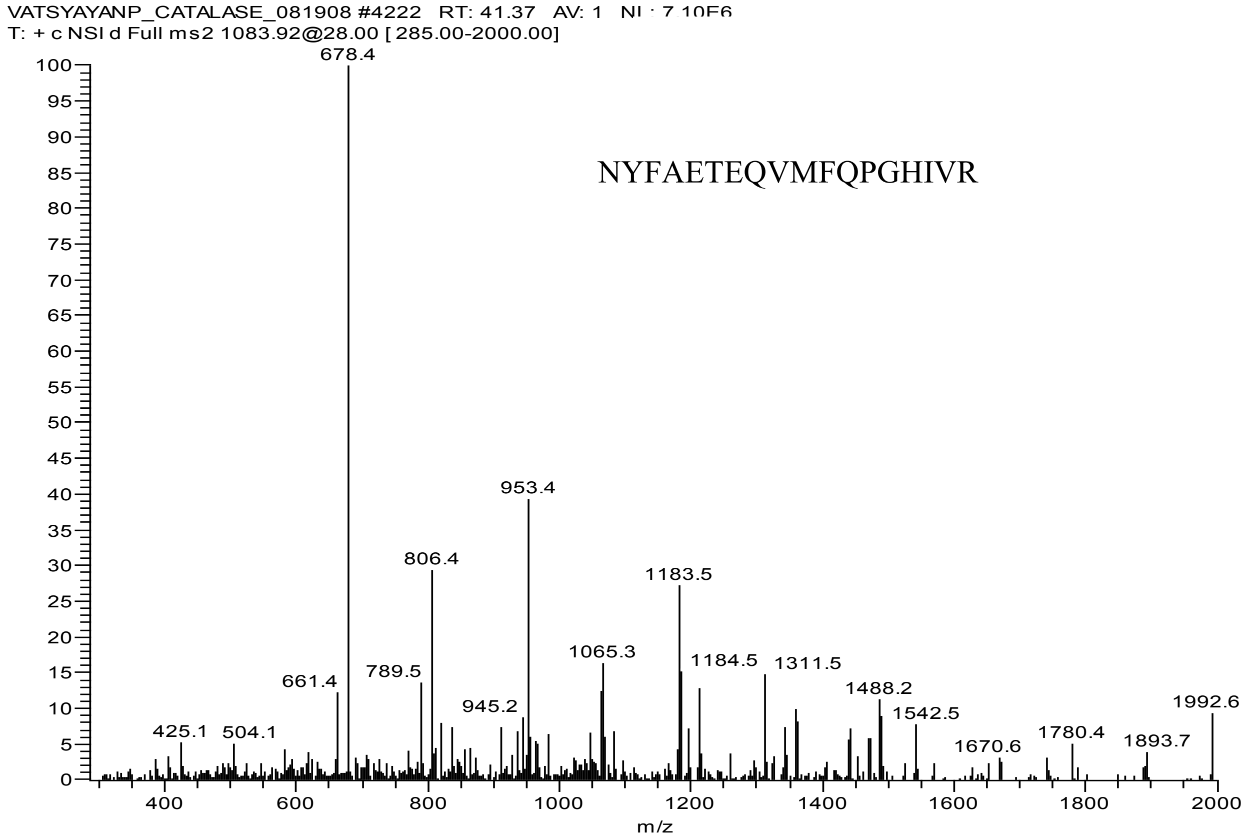

Fig. S4 LC-MS/MS tandem mass spectra of "NYFAETEQVMFQPGHIVR" peptide which belong to the tetramer interface domain of the CAT
